# FEASIBILITY OF AN AUTOMATED SELF-ADMINISTERED 24 HOUR (ASA-24) DIETARY ASSESSMENT TOOL IN OLDER ADULTS

**DOI:** 10.1093/geroni/igad104.2815

**Published:** 2023-12-21

**Authors:** Hillary Spangler, Tiffany Driesse, Michael Fowler, David Lynch, Danae Gross, Xiaohui Liang, John Batsis

**Affiliations:** UNC School of Medicine, Chapel Hill, North Carolina, United States; UNC School of Medicine, Chapel Hill, North Carolina, United States; UNC Chapel Hill, Chapel Hill, North Carolina, United States; UNC School of Medicine, Chapel Hill, North Carolina, United States; University of North Carolina, Chapel Hill, North Carolina, United States; University of Massachusetts Boston, Boston, Massachusetts, United States; UNC School of Medicine, Chapel Hill, North Carolina, United States

## Abstract

**Background:**

Certain dietary patterns impact trajectories of healthy aging. However, dietary assessment tools can be challenging to use. With the increased technology use in older adults, we aimed to explore the feasibility of completing an online, Automated Self-Administered 24-hour (ASA-24) dietary assessment tool.

**Methods:**

We conducted a randomized, crossover study of n=20 community-dwelling older adults’ (>65 years) preference for self-administered (SA) versus research assistant-administered (RAA) ASA-24. Participants were recruited via ResearchMatch.com and randomly allocated 1:1 to each group and completed either an SA or RAA ASA-24. One week later, the alternative sequence was completed. After each session, participants completed an online 11-item feasibility survey (Likert-scale;1-5, strongly disagree to strongly agree). Mean and standard deviations were reported for each question.

**Results:**

Mean age was 69±3.5 years (90% females), and no differences were observed for sex, age, race, ethnicity, education, or income. Neither group felt a need for RA assistance (p=0.34). However, both groups felt the system was easier to follow with a research assistant (RAA 4.4±1.3 vs. SA 4.6±0.5, p=0.65), particularly when self-administered first (p=0.04). When conducting an SA ASA-24 first, there was less confidence the system could be learned quickly (SA 4.5±0.5 vs RAA 3.4 ±1.0, p=0.001).

**Conclusion:**

Older adults can complete dietary recall using a self-administered online assessment; however, use of a research assistant led to better accessibility. Order of administration method may impact perceived ease of use. Next steps include increasing sample diversity and using an RAA ASA-24 platform to identify dietary patterns that impact aging.

